# Exploring supervised machine learning approaches to predicting Veterans Health Administration chiropractic service utilization

**DOI:** 10.1186/s12998-020-00335-4

**Published:** 2020-07-17

**Authors:** Brian C. Coleman, Samah Fodeh, Anthony J. Lisi, Joseph L. Goulet, Kelsey L. Corcoran, Harini Bathulapalli, Cynthia A. Brandt

**Affiliations:** 1 Pain Research, Informatics, Multimorbidities, and Education (PRIME) Center, VA Connecticut Healthcare System, 11-ACSL-G, 950 Campbell Avenue, West Haven, CT 06516 USA; 2 Yale School of Medicine, Yale University, New Haven, CT USA

**Keywords:** Machine learning, Predictive Modeling, Chiropractic, Healthcare service utilization

## Abstract

**Background:**

Chronic spinal pain conditions affect millions of US adults and carry a high healthcare cost burden, both direct and indirect. Conservative interventions for spinal pain conditions, including chiropractic care, have been associated with lower healthcare costs and improvements in pain status in different clinical populations, including veterans. Little is currently known about predicting healthcare service utilization in the domain of conservative interventions for spinal pain conditions, including the frequency of use of chiropractic services. The purpose of this retrospective cohort study was to explore the use of supervised machine learning approaches to predicting one-year chiropractic service utilization by veterans receiving VA chiropractic care.

**Methods:**

We included 19,946 veterans who entered the Musculoskeletal Diagnosis Cohort between October 1, 2003 and September 30, 2013 and utilized VA chiropractic services within one year of cohort entry. The primary outcome was one-year chiropractic service utilization following index chiropractic visit, split into quartiles represented by the following classes: 1 visit, 2 to 3 visits, 4 to 6 visits, and 7 or greater visits. We compared the performance of four multiclass classification algorithms (gradient boosted classifier, stochastic gradient descent classifier, support vector classifier, and artificial neural network) in predicting visit quartile using 158 sociodemographic and clinical features.

**Results:**

The selected algorithms demonstrated poor prediction capabilities. Subset accuracy was 42.1% for the gradient boosted classifier, 38.6% for the stochastic gradient descent classifier, 41.4% for the support vector classifier, and 40.3% for the artificial neural network. The micro-averaged area under the precision-recall curve for each one-versus-rest classifier was 0.43 for the gradient boosted classifier, 0.38 for the stochastic gradient descent classifier, 0.43 for the support vector classifier, and 0.42 for the artificial neural network. Performance of each model yielded only a small positive shift in prediction probability (approximately 15%) compared to naïve classification.

**Conclusions:**

Using supervised machine learning to predict chiropractic service utilization remains challenging, with only a small shift in predictive probability over naïve classification and limited clinical utility. Future work should examine mechanisms to improve model performance.

## Introduction

### Rationale

Chronic pain is highly prevalent and carries a high cost burden, conservatively estimated at over $560 billion annually and exceeding that of cardiovascular, neoplastic, and metabolic diseases [1]. In 2016, an estimated 20.4% of U.S. adults (50.0 million) experienced chronic pain and 8.0% (19.6 million) experienced high-impact chronic pain, with a higher prevalence in adults receiving public healthcare coverage [2].

Spinal pain conditions, including low back pain and neck pain, are among the most common musculoskeletal pain conditions and contribute greatly to the high prevalence of chronic pain, with 6.0% of U.S. adults experiencing chronic spinal pain and 2.2% experiencing high-impact chronic spinal pain [3]. Spinal pain conditions carry high spine-related and overall healthcare costs, with an average of $3915 spent on spine-related healthcare and $9781 on overall healthcare costs per chronic spinal pain patient per year [3]. High-impact chronic spinal pain carries even greater direct costs ($5979 for spine-related healthcare and $14,661 for overall healthcare). Indirect costs, including lost productivity due to disability, are also exceptionally high in this population [4].

Conservative interventions for spinal pain conditions have been associated with lower healthcare costs and improvements in pain status in different clinical populations, including veterans [5–9]. Veterans with musculoskeletal pain conditions, especially those of chronic nature, often utilize non-pharmacological pain management recommended by clinical practice guidelines, including U.S. Department of Veterans Affairs (VA) chiropractic care [10–13]. Many veterans with musculoskeletal pain conditions, including those receiving chiropractic care, demonstrate high prevalence of comorbid medical and mental health conditions [14–16], with those having higher comorbidity burdens demonstrating greater healthcare utilization [17].

Little is currently known about predicting healthcare service utilization in the domain of conservative intervention for spinal pain conditions. The proliferation of data available in the electronic health record (EHR) has led to a growing demand for prospective data-driven decision making through predictive analytics. Massive quantities of patient data are now easily accessible and rapidly queryable to enhance medical decision making, support data visualization, and create data repositories that can be used to develop predictive models [18]. The large scale of routinely collected data support the utility of predictive models compared to traditional clinical prediction rules that require parsimonious criteria, easy computability, and independent validation that may take years to complete [19]. Predictive models have demonstrated great utility and potential in many clinical disciplines where prospective prediction can inform patient management and outcomes, aid in system-level resource allocation and logistics, and afford the opportunity for cost containment [19, 20]. Data collected in EHRs can be preprocessed, mined, and subsequently support real-time, point-of-care decision making through automated processes that enable scalability across systems and clinical disciplines [21].

Predictive analytics relevant to chiropractic practice has been limited to clinical prediction rules associated with response to spinal manipulation [22, 23]. Prior studies have examined components of service utilization as “dose” and “frequency” effects of spinal manipulation, however definitions of these terms vary considerably across studies [24]. Visit frequency recommendations included in clinical practice guidelines have been largely based on Delphi panels of expert opinions [25, 26], with current evidence suggesting that spinal manipulative treatment visit frequency does not significantly impact clinical outcomes during and following the treatment period [24].

Studies have not yet examined prediction of chiropractic service utilization. VA provides an important setting to examine chiropractic service utilization as the largest integrated healthcare system in the United States with an enterprise-wide health information system supporting system-level examination of comprehensive EHR data [27]. As a capitated delivery model, VA EHR data also affords the ability to examine chiropractic service utilization as relatively independent of third-party reimbursement influence, compared to traditional delivery settings where the fee-for-service structure may confound utilization.

### Objective

Effectively predicting healthcare service utilization may help to improve care delivery and inform resource allocation. In this proof-of-concept work, we aim to explore the utility of a supervised machine learning approach in predicting one-year chiropractic service utilization by veterans receiving VA chiropractic care.

## Methods

The predictive models in this study were developed and reported in accordance with published recommendations for reporting machine learning models [28]. This study was approved by the Institutional Review Board at the VA Connecticut Healthcare System.

### Setting and dataset

The Musculoskeletal Diagnosis (MSD) Cohort, a cohort study using comprehensive national EHR data to examine musculoskeletal pain and pain care of veterans, was used as the data source for this study [14]. To be included in the MSD cohort, a veteran had to have one of 1685 International Classification of Diseases, 9th revision (ICD-9) musculoskeletal disorder diagnoses. Diagnoses had to be recorded during two or more outpatient visits within 18 months or during at least one inpatient stay. Additional sociodemographic and clinical data were extracted from the VA Corporate Data Warehouse for eligible veterans to allow for longitudinal analyses following entry into the cohort.

**Fig. 1:**
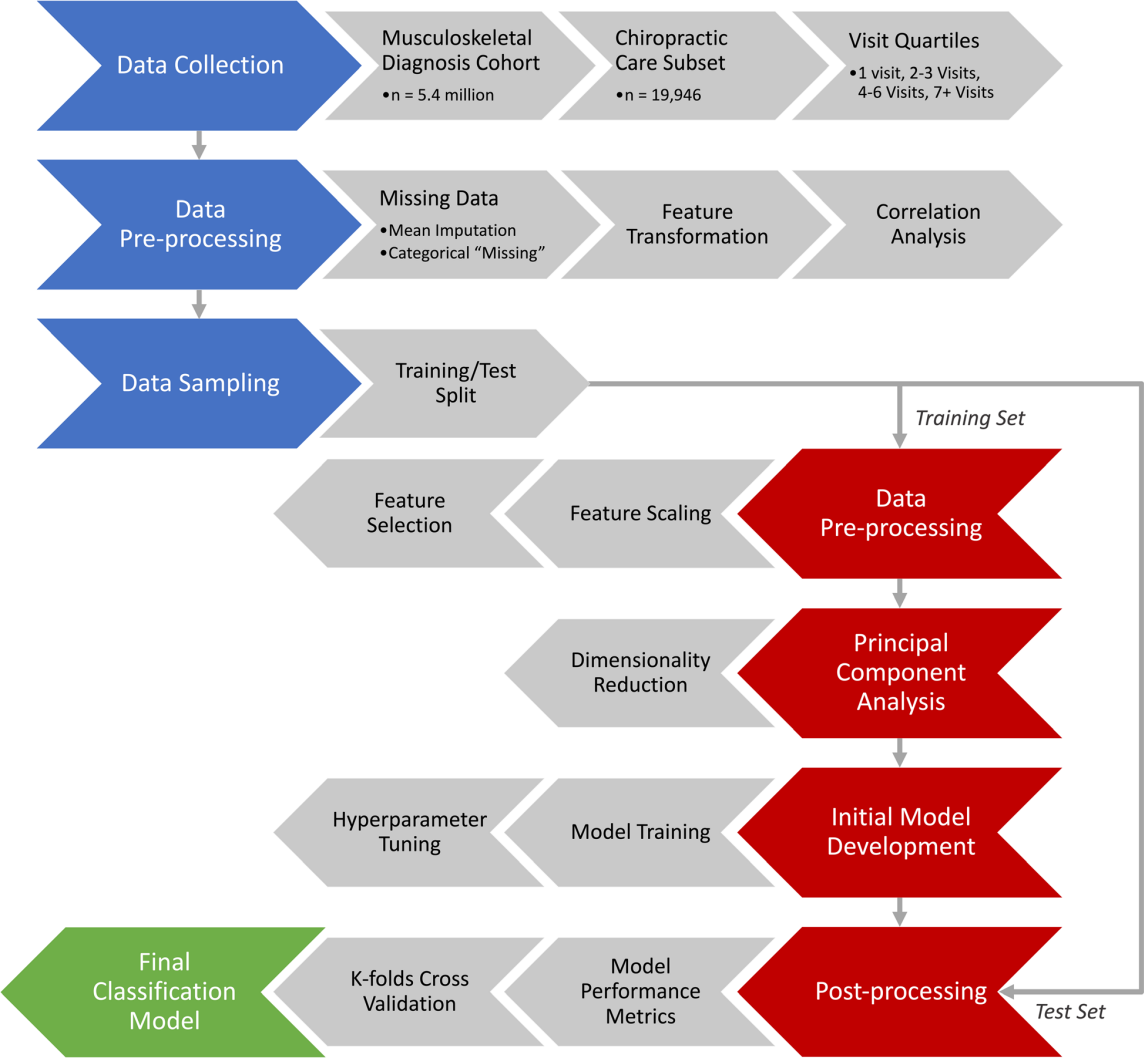
Flowchart of one-year chiropractic service utilization classification from Musculoskeletal Diagnosis Cohort data and the Chiropractic Care Subset

Figure 1 summarizes the collection, processing, and flow of data through our study. For this study, we included veterans who entered the MSD Cohort between October 1, 2003 and September 30, 2013 with at least one visit to on-station VA chiropractic services (denoted by the VA clinic stop code “436”) within 365 days of entering the MSD Cohort. This was done to ensure demographic and clinical data collected at the veteran’s MSD Cohort entry was reasonably proximate to the veteran’s first (index) chiropractic visit. One-year chiropractic service utilization was examined as the total visit frequency obtained by counting the number of visits over a period of 365 days following the veteran’s index chiropractic visit, with associated ICD-9 diagnosis codes obtained for each visit.

The diagnosis category of the index chiropractic visit was included in the final dataset. Visits were categorized as “Low back pain only”, “Neck pain only”, “Both low back and neck pain”, or “Neither low back nor neck pain” using an existing framework for identifying back and neck pain disorders in administrative data based on ICD-9 diagnoses [29].

Additional sociodemographic and clinical data were obtained for each veteran from their EHR, including cohort entry date, index chiropractic visit date, age at index chiropractic visit, index chiropractic visit facility, gender, period of service, service connected disability status, marital status, pain intensity numerical rating scale (NRS) score, body mass index (BMI), race/ethnicity, smoking status, and Charlson Comorbidity Index (CCI). Additional clinical data included binary classification of the presence of many medical comorbidities, mental health comorbidities, musculoskeletal comorbidities, and prescription data within the veteran’s VA medical record.

Statistical analyses of clinical and sociodemographic variables across independent groups used single factor ANOVA for continuous variables and chi-square tests for categorical variables, with a significance level of 0.05.

### Prediction problem

We sought to predict one-year chiropractic service utilization as a retrospective, prognostic multiclass classification problem. One-year chiropractic service utilization was categorized for each veteran into quartiles as the class label, determined by the distribution of the entire dataset. This was done to create more uniformly distributed classes, to protect against the influence of outliers showing high service utilization skewing the model, and to allow a single label, multiclass classification approach using one-versus-rest classification.

### Data preparation, feature selection, and feature engineering

Additional preprocessing was performed on the included features in the dataset. Entry year into the MSD Cohort was transformed as the number of years since 2003, the minimum entry year in this sample. The MSD Cohort entry date and the index chiropractic visit date were transformed such that the difference between the two dates (in days) was included as an engineered feature. All categorical variables were binarized into unique features using one-hot encoding. Mean imputation was used for cases with missing continuous body mass index data (*n* = 278, 1.4%). Missing categorical data were not imputed, with “Unknown” and “Unknown/Missing” as valid data entries for marital status, smoking status, and pain status.

No features demonstrated a clinical relationship or a strong correlation to one-year chiropractic service utilization, with the potential for information leakage limited. To account for collinear relationships between independent features affecting the model performance, features demonstrating a strong correlation with other features, using a Pearson correlation coefficient greater than 0.7 as the cutoff point, were dropped from the final dataset [30]. The final dataset included 158 features (Additional file 1).

For the initial phase of model development, the dataset was randomly partitioned with 70% allocated to the training set and 30% to the test set. Feature scaling was done using a standard scaler (with zero-mean and unit-variance), trained only on the training set and applied to the training and test sets. Feature selection using a filter technique based on chi-squared analyses was performed to evaluate the impact of selecting a subset of features on prediction accuracy [31].

### Principal component analysis

Principal component analysis (PCA) can be used to transform multi-dimensional data into fewer dimensions by geometrically projecting them into summary components that represent the variability, patterns, and relationships of the original features [32]. We used PCA on the scaled training set to reduce the dimensionality from 158 features into two principal components to visualize the separability of the four classes in two-dimensional space. We also explored whether PCA may be useful to reduce the dimensionality of our dataset into principal components representing the variability of our data and the predicted label to improve our classification performance. We used the proportion of total variance explained to determine how many principal components to retain, which has been recommended in exploratory analyses and establishes a predetermined threshold of proportion of total variance explained (often between 70 to 95%) [33]. We examined the total number of principal components required to reach a threshold of 70 and 95%, the upper and lower bounds of the recommended range, before determining whether to proceed with using PCA as inputs to our model.

### Selecting and building the model

As an exploratory study, we selected a subset of available multiclass one-versus-rest classifiers to evaluate performance, based on preliminary sweeping of available classifiers using Python 3.5 and the SciKit-Learn (Version 0.19.0) library [34] Four models were selected: a gradient boosted classifier, a stochastic gradient descent classifier, a linear support vector classifier, and an artificial neural network. A description of each selected model, including details on the hyperparameters and architectural parameters used in this study, is available as Additional File 2. Support-weighted precision, recall, F-measure, and subset accuracy (the percentage of total number of labels correctly predicted) were obtained for each algorithm in the initial development phase, with hyperparameters determined by grid-search and trial and error to maximize F-measure.

Using a one-versus-rest classification approach for each class (visit quartile) created four separate binary classifiers fitting one class against all other classes for each selected model. For the gradient boosted and stochastic gradient descent classifiers, the “OneVsRestClassifier” function of SciKit-Learn was used to build the series of binary classifiers. For the linear support vector classifier and multi-layered perceptron neural network, the additional function was not necessary as each has inherent multiclass capabilities using a one-versus-rest approach. As each class was represented by a single classifier (for each algorithm), this approach provided the advantage of being able to examine performance of the estimator for each class. A Precision-Recall curve (PRC) plot and the area under the PRC curve (AUC) were obtained for the binarized output of each one-versus-rest classifier to compare and evaluate performance of each algorithm for each class. The PRC was preferred to the Receiver Operating Characteristic curve, given the imbalance of predicting one quartile of the data against the remaining three [35].

Following initial development, we performed 10 cycles of 10-fold repeated, stratified cross-validation, for a total of 100 validation performances, to evaluate performance of the developed models. Feature scaling was done using a standard scaler during each validation iteration. Support-weighted precision, recall, F-measure, and subset accuracy were obtained for each iteration to compare performance metrics for each algorithm.

## Results

### Final model and performance

**Fig. 2:**
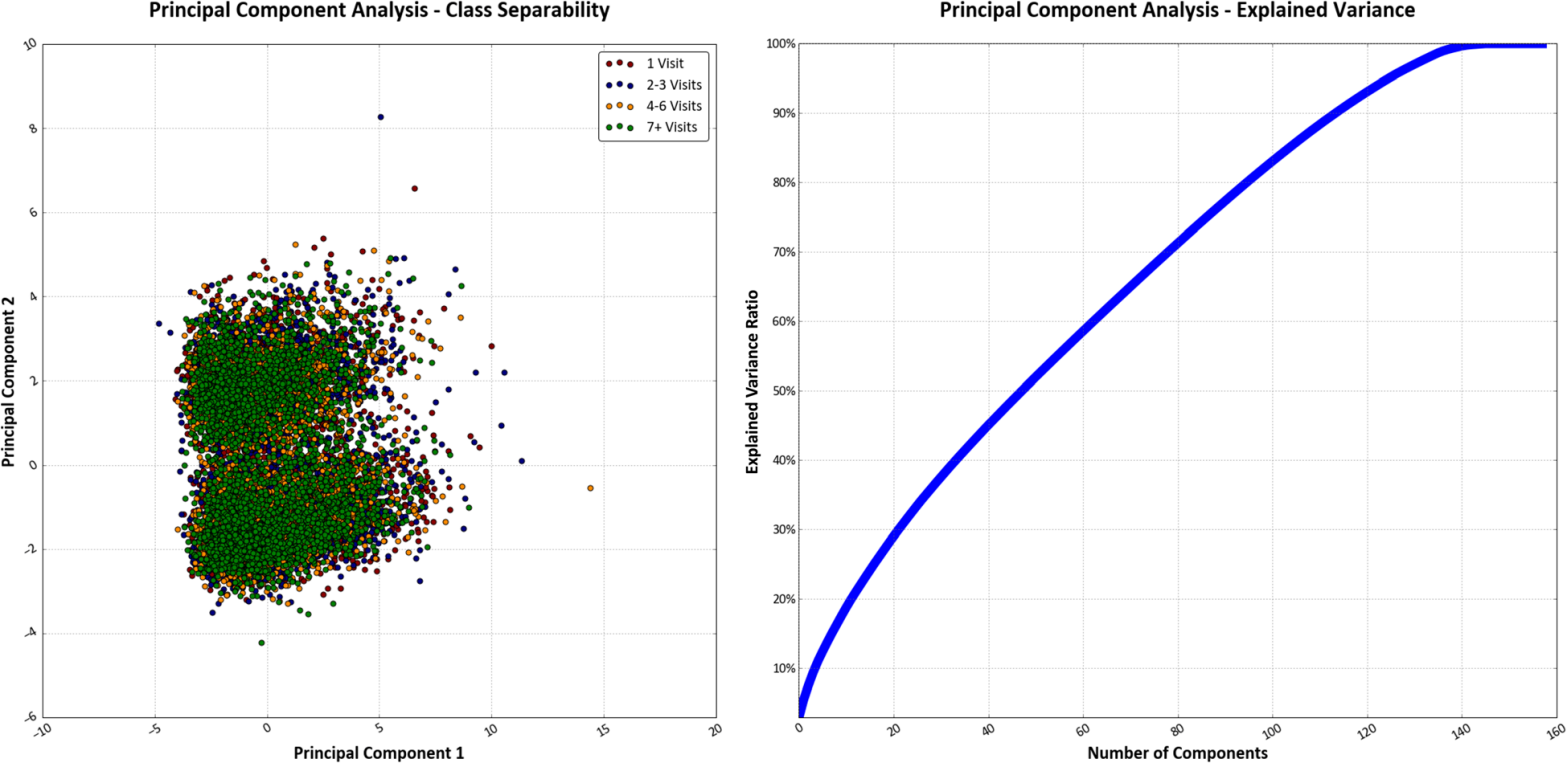
Results of principal component analysis of 158 feature inputs. Separability of four classes representing one-year chiropractic service utilization was poor when projected into two-dimensional space based on first two principal components. The explained variance ratio for the target variable as a function of the total number of principal components shows limited evidence of a strong influence on the variance between individual principal components as a predictor of the label

There were 19,946 veterans across 38 VA facilities who entered the MSD Cohort between October 1, 2003 and September 30, 2013 and had at least one visit to on-station VA chiropractic services within 1 year of MSD Cohort entry. Veteran sociodemographic and clinical characteristics are presented in Table 1. One-year chiropractic service utilization ranged from 1 visit to 73 visits and was split into quartiles representing the following classes: 1 visit, 2 to 3 visits, 4 to 6 visits, and 7 or greater visits. The distribution of classes was nearly balanced, with the first and second quartiles slightly larger than 25% of the entire dataset. Feature selection using chi-squared analyses did not affect the classifier prediction accuracy. There was poor separability of all four classes of the target variable with reduction to two-dimensions using PCA (Fig. 2). The peak explained variance ratio for an individual principal component was 2.9%, with 80 principal components required to reach a proportion of total variance explained of 70% and 126 needed to reach 95%. As such, we chose to retain all 158 features without reducing dimensionality for the purpose of predicting one-year chiropractic service utilization. We felt our sample size was sufficient to handle retaining all 158 features, given a minimum events per variable ratio of over 27:1 in predicting the smallest class.

**Table 1:** Patient sociodemographic and clinical characteristics

		Visits within 1 year				
Variable	Total	1 Visit	2–3 Visits	4–6 Visits	7+ Visits	*p* Value
N	19,946	5473 (27.4)	5233 (26.2)	4280 (21.5)	4960 (24.9)	
Age, median [IQR], y	45 [30–58]	44 [29–57]	44 [30–58]	46 [30–59]	47 [34–59]	< .00001
Sex						
*Female*	13.5	25.6	24.6	21.9	27.9	< .001
*Male*	86.5	27.7	26.5	21.4	24.4	
Index Chiropractic Visit Diagnosis						< .00001
*Low Back Pain Only*	55.6	30.5	26.3	21.1	22.2	
*Neck Pain Only*	9.0	27.6	26.3	21.6	24.5	
*Both Low Back and Neck Pain*	31.3	20.5	26.5	22.2	30.8	
*Neither Low Back nor Neck Pain*	4.1	39.1	24.0	20.4	16.4	
Race						< .00001
*White*	70.7	26.8	26.1	21.4	25.7	
*Black*	12.2	28.0	25.1	21.8	25.2	
*Hispanic*	7.7	27.0	27.8	22.7	22.5	
*Other*	2.4	26.7	23.6	24.4	25.4	
*Unknown*	7.1	33.6	28.5	19.0	18.9	
Pain intensity, median [IQR] ^a^	4 [0–6]	4 [0–6]	4 [0–6]	4 [0–6]	4 [1–7]	0.057
*No Pain or Mild Pain Intensity (NRS 0–3)*	44.3	28.8	26.6	21.2	23.4	0.190
*Moderate to Severe Pain Intensity (NRS 4–10)*	55.7	28.0	26.0	21.2	24.8	
Smoking Status ^b^						< .00001
*Never*	36.3	27.4	25.4	21.5	25.7	
*Former*	39.9	27.9	27.8	21.3	23.0	
*Current*	23.8	23.8	25.8	22.4	28.0	
BMI, mean (SD), kg/m^2 c^	29.4 (5.4)	29.1 (5.2)	29.1 (5.4)	29.1 (5.4)	29.3 (5.4)	0.054
*Not obese, BMI < 30 kg/m*^*2*^	61.6	27.5	26.1	21.8	24.6	0.311
*Obese, BMI ≥ 30 kg/m*^*2*^	38.4	27.1	26.4	20.9	25.5	
Period of Service						< .00001
*OEF/OIF/OND*	29.7	28.9	28.1	21.1	21.9	
*Gulf War*	24.3	29.1	25.7	20.8	24.4	
*Post-Vietnam Era*	12.8	25.9	25.2	22.2	26.7	
*Vietnam*	26.9	25.9	25.1	21.7	27.3	
*Other*	6.4	23.5	26.5	23.3	26.7	
Marital Status						< .01
*Married*	49.1	27.6	25.9	21.2	25.4	
*Not Married*	19.5	28.3	26.8	21.9	23.0	
*Separated/Divorced*	28.5	26.5	26.6	21.8	25.1	
*Widow/Widower*	2.3	25.3	25.8	20.9	28.0	
*Unknown*	0.5	44.3	23.6	12.3	19.8	
Service Connected Disability	65.4	26.9	25.7	21.4	26.0	< .00001
CCI, mean (SD)	0.40 (0.98)	0.36 (0.95)	0.39 (0.98)	0.40 (0.96)	0.43 (1.00)	< .01
*CCI = 0*	78.0	28.1	26.3	21.3	24.3	< .001
*CCI ≥ 1*	22.0	25.2	25.9	21.9	27.0	
Pharmaceutical Use ^d^						
*Opioid Prescription*	13.3	28.3	26.3	19.1	26.4	.010
*Tramadol Prescription*	8.0	27.8	26.9	19.5	25.8	.238
Medical Comorbidities						
*PTSD*	20.1	27.3	26.5	21.5	24.7	.967
*Mild Depression*	21.3	26.2	25.9	20.5	27.5	< .0001
*Major Depression*	7.9	24.7	26.1	22.8	26.4	.051
*Schizophrenia*	0.5	27.9	29.8	15.4	26.9	.477
*Bipolar*	4.4	24.7	28.8	21.4	25.1	.199
*TBI*	4.7	29.4	28.2	21.9	20.6	.017
*Alcohol or Substance Use Disorder*	10.7	27.1	27.4	21.0	24.6	.663

*BMI* Body mass index; *IQR* Interquartile range; *NRS* Numerical rating scale; *CCI* Charlson Comorbidity Index; *PTSD* Post-traumatic stress disorder; *TBI* Traumatic brain injury; Significance at α = 0.05; ^a^ 3621 other/missing; ^b^ 685 other/missing; ^c^ 278 missing; ^d^ Prescription within 30 days of MSD Cohort entry

Performance metrics by class for each algorithm in the initial development phase are presented in Table 2. Overall performance of the four models was poor, with no model able to predict one-year chiropractic service utilization with a support-weighted subset accuracy greater than 42.1%. Precision, recall, and F-measure were similarly generally poor across all classes in all models during initial development.

**Table 2:** Classification matrix and subset accuracy of machine learning models to predict one-year chiropractic service utilization, based on parameters from initial development phase

Model/Class	Precision (%)	Recall (%)	F-measure (%)	Accuracy (%)
**Gradient Boosted Classifier**				
*1 Visit*	43.5	61.7	51.0	
*2–3 Visits*	36.3	32.5	34.3	
*4–6 Visits*	34.0	9.2	14.5	
*7+ Visits*	46.2	57.8	51.0	
*Average*^a^	40.3	42.1	39.1	42.1
**Stochastic Gradient Descent Classifier**				
*1 Visit*	43.9	53.1	48.1	
*2–3 Visits*	32.3	29.9	31.0	
*4–6 Visits*	24.9	14.8	18.6	
*7+ Visits*	43.4	51.2	47.0	
*Average*^a^	36.8	38.6	37.2	38.6
**Support Vector Classifier**				
*1 Visit*	42.6	60.8	50.1	
*2–3 Visits*	35.3	26.1	30.0	
*4–6 Visits*	32.0	11.3	16.7	
*7+ Visits*	45.3	60.3	51.7	
*Average*^a^	39.2	41.4	38.4	41.4
**Artificial Neural Network**				
*1 Visit*	42.9	56.2	48.7	
*2–3 Visits*	35.9	30.6	33.1	
*4–6 Visits*	25.3	12.1	16.4	
*7+ Visits*	45.1	55.9	49.9	
*Average*^a^	38.0	40.3	38.2	40.3

^a^Support-weighted average

**Fig. 3:**
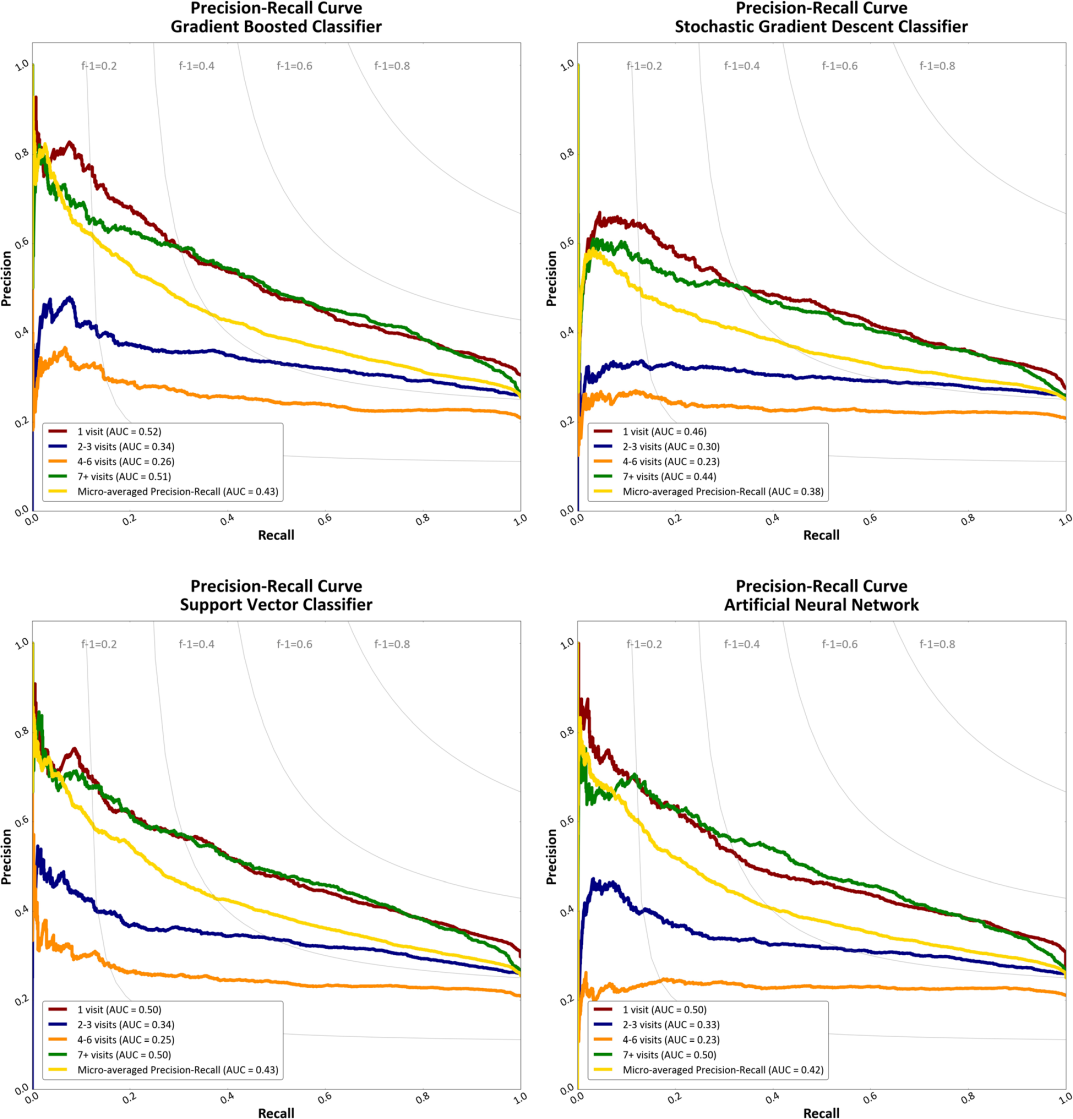
Precision-Recall curves (with area under the curve values) for the gradient boosted classifier, stochastic gradient descent classifier, support vector classifier, and artificial neural network. The iso-F-Measure curves represent the function along which all F-measure scores are equal for a given precision/recall pair

The PRC and AUC for each one-versus-rest classifier in each model is presented in Fig. 3. Each classifier was better at identifying service utilization in the first or fourth quartile (1 visit or 7 or greater visits) than those within in the interquartile range. The range of the AUC for the micro-averaged PRC was 0.38 to 0.43. Given the baseline probability of a positive outcome in each one-versus-rest classifier of approximately 25%, this represents a small positive shift in prediction probability (approximately 15%) compared to naïve classification.

**Fig. 4:**
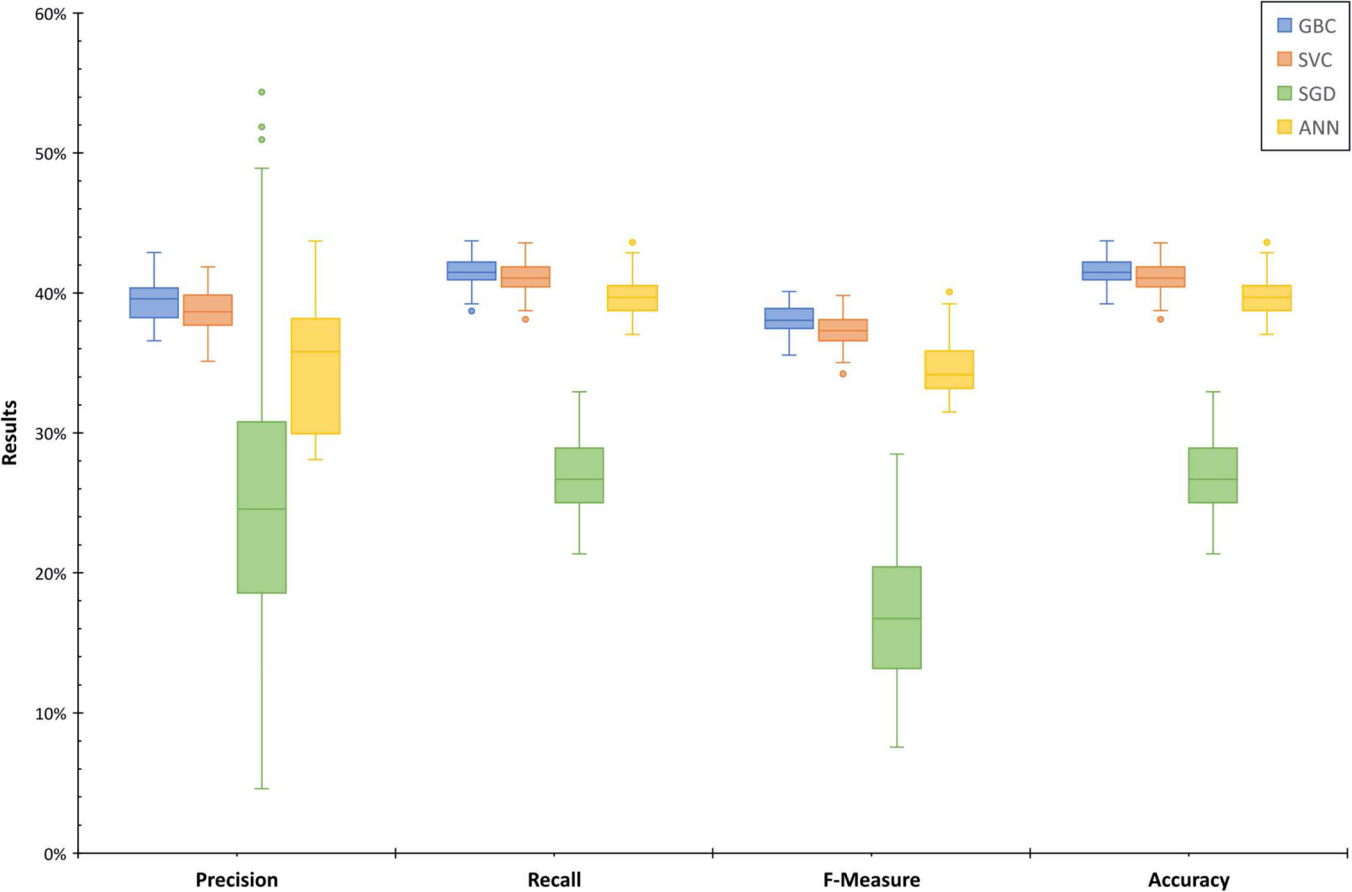
Performance metrics for cross-validation of machine learning models (GBC = Gradient boosted classifier; SVC = Support vector classifier; SGD = Stochastic gradient descent classifier; ANN = Artificial neural network) to predict one-year chiropractic service utilization. Measures are support-weighted averages of four classes across 100 iterations (using 10 replications of 10-fold cross validation)

In the cross-validation phase, we found similar performance results (Fig. 4). The gradient boosted classifier, the support vector classifier, and the artificial neural network performed most consistently (median accuracy 41.5%, 41.1, and 39.7%, respectively). The stochastic gradient descent classifier performed most inconsistently, with the largest tails. The mean precision (with 95% confidence interval) was 39.4 ± 0.3% for the gradient boosted classifier, 24.8 ± 2.0% for the stochastic gradient descent classifier, 38.7 ± 0.3% for the support vector classifier, and 34.5 ± 0.9% for the artificial neural network. The mean recall (with 95% confidence interval) was 41.5 ± 0.2% for the gradient boosted classifier, 26.8 ± 0.5% for the stochastic gradient descent classifier, 41.1 ± 0.2% for the support vector classifier, and 39.7 ± 0.2% for the artificial neural network. The mean F-measure (with 95% confidence interval) was 38.1 ± 0.2% for the gradient boosted classifier, 16.9 ± 1.0% for the stochastic gradient descent classifier, 37.3 ± 0.2% for the support vector classifier, and 34.6 ± 0.4% for the artificial neural network. The mean accuracy (with 95% confidence interval) was 41.5 ± 0.2% for the gradient boosted classifier, 26.8 ± 0.5% for the stochastic gradient descent classifier, 41.1 ± 0.2% for the support vector classifier, and 39.7 ± 0.2% for the artificial neural network.

## Discussion

Effectively predicting healthcare service utilization has multiple clinical implications and can help to improve delivery, population health, and resource allocation to support the Quadruple Aim and support transitions towards value-based care delivery systems [36, 37]. Predictive models have previously been developed and validated to predict healthcare resource utilization using patient-level EHR data [37–39]. Further, they have demonstrated outperformance of existing clinical prediction rules, with machine learning models demonstrating small performance benefits (with limited clinical differences) over statistical models [38].

Current evidence to predict the use of chiropractic services over a discrete period of time is limited. In this study, we developed four machine learning models using a large cohort of veterans receiving VA chiropractic services. While we establish a baseline by which future models may be evaluated as a proof-of-concept work, the clinical utility of these models, based on the data used in this study, may be limited at this time.

Our models yield a small positive shift in predictive probability over naïve classification but remain limited by the high amount of false positives and false negatives. Given the average precision (positive predictive value), less than half of those that are predicted in a certain class are truly in that class, resulting in many false positives. Based on the average recall (sensitivity), less than half of those that are truly in a class are correctly classified as such, resulting in many false negatives.

Difficulty in accurately predicting one-year chiropractic service utilization could be expected based on the existing body of literature and the correlation analysis performed during this study. Machine learning using complex pain-related data may help to identify phenotypic subsets of pain presentations and uncover previously unidentifiable relationships between important variables, including factors related to pain-related healthcare service utilization [40]. However, clinical or sociodemographic features correlating with higher or lower service utilization have yet to be demonstrated quantitatively and empirically. In support of this, we found no features demonstrating a strong correlation to one-year chiropractic service utilization. It is likely that the features included in this dataset may be poor predictors of service utilization and more potentially relevant data may exist, such as facility or clinic characteristics, to yield more accurate predictive capabilities. For instance, a short supply of chiropractic appointments available may impose ceiling limits on the number of visits available to a given patient, irrespective of any optimal amount. If availability were uniform across all sites and all time points, then it is possible the features included may have different predictive abilities. We find that while there may be limited explanatory value in our included variables with respect to pattern recognition of one-year chiropractic service utilization, there may be additional explanatory value of these variables with respect to recognition of other clinically meaningful patterns which warrant future investigation.

Although limited evidence currently exists to suggest a relationship between optimal amount of chiropractic care and clinical outcomes, the clinical implications of label misclassification may result in over- or under-estimation of service utilization, with both yielding potentially increased front-end or back-end system burden. Overestimating service utilization may result in decreased access to clinically indicated care for other veterans due to an overburdening of the system on the front-end through inappropriate resource allocation. Further, high rates of service overuse may substantially contribute to higher healthcare spending and may result in harms to all stakeholders in the healthcare system, especially patients [41]. Underestimating service utilization may result in an individual veteran failing to receive an adequate amount of chiropractic care that may be clinically indicated. This may cause an increase in back-end system burden in that the individual veteran may require additional resource allocation than previously predicted.

One strength of this study is that it uses one of the largest cohorts of patients receiving chiropractic care within a capitated healthcare system. We also examine the use of chiropractic services on a rolling basis over a twelve-year study period, allowing for analyses that reflect the development of VA on-station chiropractic clinics over time.

One of the most interesting findings of this study is the distribution of the chiropractic service utilization into quartiles. While empirical evidence regarding optimal treatment trial duration is limited, the recommended chiropractic treatment frequency and duration for VA patients with spine-related symptoms is up to 10 visits for uncomplicated acute episodes and up to 12 visits for complicated acute episodes and chronic conditions, based on Delphi consensus processes [26]. These data suggest that VA chiropractors have been providing care consistent with these recommendations.

For the 25% of veterans receiving 7 or more chiropractic care visits within 1 year, it is unclear if these visits were related to a single episode of care for a condition or related to a multiple episodes of care for either a new condition or reactivation of an existing condition throughout the one-year period. This quartile included the widest spread of visits (7 to 73 visits), with less than 1% of all patients receiving more than 24 visits. It is possible that patients with greater service use are of higher medical complexity with higher rates of medical and mental health comorbidities. This is consistent with previous work demonstrating higher service utilization associated with higher comorbidity burdens in the veteran population [17]. These features of complexity may be represented in our dataset, thus contributing to our models for slightly improved pattern recognition in the class of highest service use. Further, there may be a time-dependent underlying relationship between service utilization and specific facilities related to their individual clinic characteristics. Some facilities may have greater capacity for clinic visits based on clinic characteristics such as physical space and number of chiropractors, which could have a greater influence on chiropractic service utilization than patient characteristics. Additionally, provider-based factors beyond the scope of this project – such as the influence of job performance metrics and/or chiropractic practice preferences – may impact the number of visits that patients receive.

Additionally, 25% of veterans received only a single visit of VA chiropractic services. This may be confounded due to inappropriateness of chiropractic care for the veteran’s presenting condition, veteran’s preference to not seek additional chiropractic care, or a single on-site consultation followed by referral for purchased care off-site [10]. The clinical determination of inappropriateness of chiropractic care and the relationship between supply of chiropractic services by facility may be related to features included in our dataset, thus supporting pattern recognition for this class of lowest service use.

There are several limitations to this study. First, this study was performed to examine VA chiropractic service utilization specifically, which may limit generalizability to chiropractic clinics outside of VA. We used clinical data from the fully-integrated VA EHR that may not be easily obtained in private practice chiropractic clinics.

We relied on administrative data from the VA EHR as an abstraction of clinical data. We did not seek to examine what occurred at individual chiropractic visits, which may have impacted our findings. More detailed analyses of visits, including natural language processing of progress note documentation, critical evaluation of treatments provided, and inclusion of data from valid and reliable patient-reported outcome measures may provide more detailed clinical data and improve future predictive models.

We did not examine any differences in service utilization based on specific pain presentation because a substantial majority of patients in our dataset (86%) received care for low back pain alone or in combination with neck pain. We included concurrent neck pain and concurrent other musculoskeletal pain at both the index chiropractic visit and across the one-year observation period as features in our model to account for potential mediation of these on service utilization. We hypothesize little change in our classification performance based on these factors alone. Currently, there is limited literature available regarding the optimal frequency and duration of chiropractic care recommended for these specific pain presentations [24], making it unlikely that there are system-wide patterns in chiropractic service utilization based on pain presentation.

The sociodemographic and clinical data used for each veteran was based on those collected at the time of his or her MSD Cohort entry, with comorbidity diagnoses occurring from 12 months prior to 6 months after cohort entry. By limiting inclusion criteria to veterans with an index chiropractic date within 365 days of cohort entry, we attempted to limit potential inaccuracy of these data. However, it remains possible that a veteran’s health status may have changed over the 365 days following cohort entry and/or the 365 days following the index chiropractic visit. It is also possible a veteran may have presented for his or her index chiropractic visit within 6 months after cohort entry and prior to a diagnosis of a comorbidity.

We aimed to predict visit quartile as a multiclass classification problem, with our results suggesting limited clinical utility to this approach. Different results may be found by structuring the question as a binary classification problem (for example, classifying patients based on a certain clinically relevant threshold of visits) or as a regression problem (predicting service utilization across a continuous quantity of visits). We also used a sampling of commonly used classification models to predict one-year chiropractic service utilization, with a trial-and-error grid-search approach to hyperparameter tuning. It is possible, although we suspect minimally likely, that other functions and/or hyperparameters may yield stronger classification performance using these same data.

We selected a 70–30% training-testing split for our initial model development. It is possible that different results may be found by training on a larger proportion of the dataset (i.e. 80% or 90%) with a smaller testing set. However, given the similar results of our 10-fold cross validation (with a 10% testing set for each fold), increasing the size of our training set is unlikely to strongly change our classification performance.

Specific to the algorithms selected, we identified a warning in the executed Python code that was present in both the stochastic gradient descent classifier and the artificial neural network. During the cross-validation phase, both algorithms resulted in zero instances of a predicted class during a small number of iterations. We recognize this as a limitation in spite of using 10-fold repeated, stratified cross validation, with it possible that an individual class may not be predicted in some iterations of these models. Additional calibration of these models to better predict labels more consistent with baseline probabilities may help to address this. This may have contributed to the weaker and less consistent performance of these two models compared to the gradient boosted classifier and support vector classifier.

## Conclusion

Overall, we have demonstrated that using supervised machine learning to predict chiropractic service utilization remains challenging. Preliminary performance shows a small shift in predictive probability over naïve classification. However, model performance metrics suggest limited clinical utility at this time based on the features included in our dataset. Future work should examine mechanisms to improve model performance, including collecting potentially more relevant data such as facility and clinic access characteristics, progress note documentation, treatments rendered, and patient-reported outcome measures.

## Supplementary information

**Additional file 1. **Variables included in the final dataset.

**Additional file 2.** Detailed description of methods, including machine learning algorithms used.

## Data Availability

To maximize protection security of veterans’ data while making these data available to researchers, the US Department of Veterans Affairs (VA) developed the VA Informatics and Computing Infrastructure (VINCI). VA researchers must log onto VINCI via a secure gateway or virtual private network connection (VPN), and use a virtual workspace on VINCI to access and analyze VA data. By VA Office of Research and Development policy, VINCI does not allow the transfer of any patient-level data out of its secure environment without special permission. Researchers who are not VA employees must be vetted and receive “without compensation” (WOC) employee status to gain access to VINCI. All analyses performed for this study took place on the VINCI platform. For questions about data access, contact the study lead (Brian.Coleman2@va.gov) or the VA Office of Research and Development (VHACOORDRegulatory@va.gov).
