# Supplementary material for: Exploring supervised machine learning approaches to predicting Veterans Health Administration chiropractic service utilization
Source: Chiropr Man Therap. 2020 Jul 17;28:47. doi: 10.1186/s12998-020-00335-4 (PMC7368704; doi:10.1186/s12998-020-00335-4)
Supplement: Supplementary file 1 — Additional file 1. Variables included in the final dataset. [file 12998_2020_335_MOESM1_ESM.docx]

**SUPPLEMENTAL MATERIAL.** Detailed description of selected machine learning models.

In this supplemental material, we describe the four selected machine learning models that were used in this study: a gradient boosted classifier, a stochastic gradient descent classifier, a linear support vector classifier, and an artificial neural network. Python 3.5 and the SciKit-Learn (Version 0.19.0) library^1^ were used to implement these models, with the default hyperparameters used for each model, except as noted based on a modified pipeline using grid-searching and to establish a random state for all models. Our subset was derived from the larger list of multiclass classification models available in the SciKit-Learn library, based on a rapid, preliminary sweep of model performance on our dataset.

Gradient boosted classification fits an additive model in a forward stage-wise manner.^2-4^ For each stage, a new “weak learner” (decision tree) is incorporated into the model to update and adjust the model’s current state based on a negative gradient determined by the loss function, until convergence or an upper iteration limit is reached. The goal of this process remains to minimize the loss function (a measure of how well the function’s coefficients represent the data) and, ideally, match the predicted probability distribution as closely as possible to the true probability distribution. Within the SciKit-Learn gradient boosted classifier algorithm, the default loss function is the binomial deviance loss function, which lends well to predicting binary classification problems. While the classification problem is multiclass, using one-vs-rest classification created a series of 4 binary classifiers. The default number of boosting stages to perform was 100 stages, with a default minimum number of samples to split at an internal node as 2.

Stochastic gradient descent classification is a popular method for large scale supervised machine learning problems.^5,6^ While the gradient boosted method adds weak learners at each stage to build a final ensemble model that optimizes the composite model prediction, gradient descent methods computes the next set of model parameters to optimize the loss function of a static model architecture.^7^ Given our sample size, we expected that the stochastic (random) nature of sampling would be more suitable than batch gradient descent methods (using the entire sample) with respect to computational efficiency. A logistic loss function was used in this model rather than the default hinge loss function (which would give a linear support vector machine). The “penalty term” used was the default L2 (squared Euclidean norm) regularization term to shrink model parameters towards a zero vector. The constant multiplied by the regularization term, alpha, was determined by trial and error with alpha $\in$ [0.1, 0.01, 0.001, 0.0001], and set to 0.01 to maximize the F-measure.

Support vector classification aims to draw a decision boundary (or hyperplane) to separate two classes in space that maximizes the distance between the hyperplane and the nearest points to the linear boundary.^8,9^ This results in only a subset of all points (called support vectors) contributing to the position decision function. The boundary function is determined by the algorithm’s kernel and may be represented by a linear function, polynomial function, radial-based function, among others (including a custom function). Multiclass support vector classification identifies a series of functions whose shape is determined based on the kernel to define one or more hyperplanes to separate classes in partitions of multidimensional space. We used a linear support vector classifier model, which uses the linear kernel to create four linear hyperplanes separating the data into each of the four classes, with a squared hinge loss function. To address the duality of the optimization problem,^10^ the algorithm was set to solve the primal optimization problem (based on the sample size being much larger than the number of features) with the dual parameter set to “False”.

Artificial neural networks enable a form of deep learning in which a model learns complex and highly-nonlinear relationships between feature inputs and class outputs through a series of layers of interconnected neurons.^11^ The input layer consists of a set of neurons representing the input features from the dataset. In a multilayer neural network, subsequent layers known as hidden layers are built with a unique architecture of additional neurons and can be used to create more powerful networks than a single-layer network.^12^ Determining the number of hidden layers and number of neurons in a hidden layer is often dependent on the individual application, with the trial and error method most often employed to determine the optimal architecture.^13^ The neural network architecture ultimately connects to a final output layer, consisting of a set of neurons that represent each class, allowing for inherent multiclass classification. The multilayer perceptron neural network used included 158 neurons in the input layer (one for each feature), 4 neurons in the output layer (a Softmax layer, with one neuron for each class), and a single hidden layer with 5 neurons determined by trial and error using a range of 1 to 10 neurons in 1 to 5 hidden layers and maximizing the F-measure. The default hyperparameters were used for the model, including the “Adam” optimization solver^14^ and a maximum number of 200 iterations before convergence.

**References**

1. Pedregosa FV, G.; Gramfort, A.; Michel, V.;Thirion, B.; Grisel, O.; Blondel, M.; Prettenhofer, P.; Weiss, R.; Dubourg, V.; Vanderplas, J.; Passos, A.; Cournapeau, D.; Brucher, M.; Perrot, M.; Duchesnay, E. Scikit-learn: Machine Learning in Python. *Journal of Machine Learning Research*. 2011;12:2825-30.

2. Friedman JH. Greedy function approximation: A gradient boosting machine. *Ann Statist*. 2001;29:1189-1232.

3. Natekin A and Knoll A. Gradient boosting machines, a tutorial. *Front Neurorobot*. 2013;7:21-21.

4. Luna JM, Gennatas ED, Ungar LH, Eaton E, Diffenderfer ES, Jensen ST, Simone CB, 2nd, Friedman JH, Solberg TD and Valdes G. Building more accurate decision trees with the additive tree. *Proc Natl Acad Sci U S A*. 2019;116:19887-19893.

5. Zhang T. Solving Large Scale Linear Prediction Problems Using Stochastic Gradient Descent Algorithms. *ICML 2004: Proceedings of the Twenty-First International Conference of Machine Learning*. 2004:919-926.

6. Bottou L. Large-Scale Machine Learning with Stochastic Gradient Descent. *Proceedings of the 19th International Conference on Computational Statistics (COMPSTAT'2010)*. 2010:177-187.

7. Parr T and Howard J. Gradient boosting performs gradient descent. 2019.

8. Manning CD, Raghavan P and Schutze H. *Introduction to Information Retrieval*: Cambridge University Press; 2008.

9. Xu Y, Shao Y, Tian Y and Deng N. Linear Multi-class Classification Support Vector Machine. *Cutting-Edge Research Topics on Multiple Criteria Decision Making*. 2009:635-642.

10. Bradley SP, Hax AC and Magnanti TL. Duality in Linear Programming *Applied Mathematical Programming*: Addison-Wesley; 1977: 130-174.

11. Demirci F, Akan P, Kume T, Sisman AR, Erbayraktar Z and Sevinc S. Artificial Neural Network Approach in Laboratory Test Reporting:  Learning Algorithms. *American Journal of Clinical Pathology*. 2016;146:227-237.

12. Hagan MT, Demuth HB, Beale MH and De Jesus O. *Neural Network Design.* 2 ed; 2014.

13. Sheela KG and Deepa SN. Review on Methods to Fix Number of Hidden Neurons in Neural Networks. *Mathematical Problems in Engineering*. 2013;2013:11.

14. Kingma DP and Ba J. Adam: A Method for Stochastic Optimization. *arXiv e-prints*. 2014.
